# Human immunodeficiency virus in institutionalized elderly people: Vírus da imunodeficiência humana em idosos institucionalizados

**DOI:** 10.1590/1516-3180.2016.0034150516

**Published:** 2016-10-13

**Authors:** Milton Luiz Gorzoni, Sueli Luciano Pires, Lilian de Fátima Costa Faria, Márcia Regina Valadares Aguado, Miriam Carmen Santana

**Affiliations:** I MD, PhD. Associate Professor, Faculdade de Ciências Médicas da Santa Casa de São Paulo (FCMSCSP), and Head of Area III of the Department of Internal Medicine and Coordinator of the Geriatrics Sector, Irmandade da Santa Casa de Misericórdia de São Paulo, São Paulo, SP, Brazil.; II MD, MSc. Instructor, Faculdade de Ciências Médicas da Santa Casa de São Paulo (FCMSCSP), and Technical Director, Hospital Geriátrico e de Convalescentes D. Pedro II, Irmandade da Santa Casa de Misericórdia de São Paulo, São Paulo, SP, Brazil.; III MD, MSc. Attending Physician, Hospital Geriátrico e de Convalescentes D. Pedro II, Irmandade da Santa Casa de Misericórdia de São Paulo, São Paulo, SP, Brazil.; IV RN. Nurse, Hospital Infection Control Commission, Hospital Geriátrico e de Convalescentes D. Pedro II, Irmandade da Santa Casa de Misericórdia de São Paulo, São Paulo, SP, Brazil.; V RN. Nurse, Hospital Geriátrico e de Convalescentes D. Pedro II, Irmandade da Santa Casa de Misericórdia de São Paulo, São Paulo, SP, Brazil.

**Keywords:** Aged, Health services for the aged, HIV seroprevalence, HIV infections, Homes for the aged, Idoso, Serviços de saúde para idosos, Soroprevalência de HIV, Infecções por HIV, Instituição de longa permanência para idosos

## Abstract

**CONTEXT AND OBJECTIVE:**

A search in the SciELO and PubMed databases showed few studies on human immunodeficiency virus (HIV) positive individuals in long-term care institutions (LTCIs), thus prompting the present study. The aim of this study was to ascertain whether there were any HIV-positive individuals in LTCIs for the elderly.

**DESIGN AND SETTING:**

Cross-sectional study in which the Hospital Infection Control Committee (HICC) of a 405-bed LTCI was consulted.

**METHODS:**

The medical records of 405 individuals interned in the LTCI who had been tested for HIV infection were requested for analysis of the following variables: [1] age and gender; [2] length of stay at LTCI (months); [3] causes and diagnoses on admission to LTCI according to International Classification of Diseases, 10^th^ edition; [4] date of HIV diagnosis; [5] seropositivity for syphilis and hepatitis B and C viruses; [6] medications used at last prescription in medical file; and [7] mean CD4 lymphocyte count based on: total lymphocyte count/6 and total lymphocyte count x 0.8 x 0.2 or 0.3.

**RESULTS:**

Four men were HIV-positive, with mean age 71.2 ± 8.6 years, LTCI stay 74.2 ± 38.1 months and length of HIV diagnosis 24.5 ± 17 months (confirmed by HICC standard screening). Three had stroke sequelae; one, dementia syndrome; two, seropositivity for syphilis; two, hepatitis B and one, hepatitis C. The main drugs used were lamivudine, zidovudine, lopinavir, ritonavir, levothyroxine, omeprazole, ranitidine, lactulose and risperidone. The estimated CD4 count was 341 ± 237/mm^3^.

**CONCLUSIONS:**

HIV-positive individuals are present in LTCIs, diagnosable through serological screening and treatable with antiretroviral drugs.

## RESUMO

**CONTEXTO E OBJETIVO:**

Busca nos portais SciELO e PubMed encontrou poucos estudos sobre indivíduos positivos para o vírus da imunodeficiência humana (HIV) em instituições de longa permanência para idosos (ILPIs), fato este que justifica o presente estudo. O objetivo foi verificar se há soropositivos para o HIV em instituições de ILPIs.

**TIPO DE ESTUDO E LOCAL:**

Estudo transversal por consulta à Comissão de Controle de Infecção Hospitalar (CCIH) de ILPI com 405 leitos.

**MÉTODOS:**

Solicitaram-se 405 prontuários de pacientes internados, nos quais se pesquisou sorologia reagente ao HIV para análise de: [1] idade e gênero; [2] período de internação na ILPI (meses); [3] causa(s) e diagnósticos à internação na ILPI pela Classificação Internacional de Doenças, 10^a^ edição; [4] data do diagnóstico do HIV; [5] soropositividade para sífilis e vírus da hepatite B e C; [6] medicamentos em uso na última prescrição no prontuário; e [7] média de linfócitos CD4 baseada em: número total de linfócitos/6 e número total de linfócitos x 0,8 x 0,2 ou 0,3.

**RESULTADOS:**

Quatro homens eram HIV-positivos. Eles tinham 71,2 ± 8,6 anos de idade; 74,2 ± 38,1 meses na ILPI e 24,5 ± 17 meses de soropositividade (diagnósticos realizados como triagem padrão da CCIH). Havia sequelas de acidente vascular cerebral em 3 e síndrome demencial em 1; sorologias positivas para sífilis em 2, vírus hepatite B em 2 e C em 1. Os principais fármacos utilizados eram: lamivudina, zidovudina, lopinavir, ritonavir, levotiroxina, omeprazol, ranitidina, lactulona e risperidona. O CD4 foi estimado em 341 ± 237/mm^3^.

**CONCLUSÕES:**

Há soropositivos para o HIV em ILPIs, passíveis de diagnóstico em triagens sorológicas e de tratamento com antirretrovirais.

## INTRODUCTION

Elderly people can be defined as individuals with a chronological age of 60 years or over.^1^ This age group is currently growing worldwide and particularly in Brazil.^2^ The aging process of this population also creates an increasing need for long-term care.^3^ Long-term care institutions (LTCIs) have their own dynamics and provide care for specific subpopulations among the elderly.^4^ The question of whether these subpopulations include individuals infected with the human immunodeficiency virus (HIV) arises.

The first case report on acquired immunodeficiency syndrome (AIDS) in Brazil was published in 1980.^5^ It involved a retrospective diagnosis on a young bisexual adult living in the state of São Paulo.^5^ Cases of AIDS among individuals aged 60 years and older were first reported in 1984, reaching 13,657 cases by mid-2009.^6^ This number accounted for 2.5% of all cases of AIDS diagnosed between 1980 and 2009 in Brazil.^6^ During this period, the first case of AIDS in an elderly person at the Irmandade da Santa Casa de Misericórdia de São Paulo hospital was reported.^7^ Curiosity in relation to this occurrence led us to look more deeply into the topic. Interestingly, we found nearly nothing regarding HIV-positive patients in LTCIs.

It is noteworthy that following the advent of combination antiretroviral therapy and free provision to all HIV-positive Brazilians, the survival of these individuals in Brazil has increased, thus leading to greater numbers of patients aged 50 years or over.^6^ Nevertheless, there remains an absence of specific therapeutic guidelines for HIV among the elderly, despite the higher rates of side effects and drug-drug interactions in this group. Moreover, the clinical condition relating to the process of human aging associated with HIV infection remains undefined.^6^^,^^8^^,^^9^

The questions that can thus be posed are: Is the same demographic process occurring among residents in LTCIs? Are there any HIV-positive LTCI residents requiring diagnosis and specific care? A search conducted in the websites http://www.scielo.br/ and http://www.nlm.nih.gov/ on January 12, 2014, found very little information on HIV-positive LTCI residents.^10^^,^^11^ This finding prompted the present study, in view of the sparseness of the literature on HIV-positive LTCI residents.

## OBJECTIVE

The aim of this study was to ascertain whether there were any HIV-positive individuals in LTCIs for the elderly.

## METHODS

### Design and setting

This was a cross-sectional study based on medical records, conducted through the Hospital Infection Control Committee (HICC) at a 405-bed LTCI belonging to a philanthropic institution. This LTCI is affiliated to an undergraduate medical course and to medical residency programs in the city of São Paulo. This institution was chosen because of its large number of patients, its academic affiliation and its HICC admission protocol of actively seeking potentially serious diseases among residents that would pose a possible risk of contamination to healthcare professionals who come into contact with these individuals.

Surveillance swab specimens were collected to test for *Klebsiella pneumoniae* carbapenemase-producing bacteria and serological tests for syphilis, HIV and hepatitis B and C viruses were performed. New admissions were also assessed with regard to active tuberculosis, scabies, pediculosis and diarrhea-related diseases.

The medical records of the HIV-positive residents were requested for analysis of the following variables:

1. age and gender;
2. length of stay at the LTCI in months;
3. causes and diagnoses on admission to the LTCI according to the International Classification of Diseases, 10^th^ edition;^12^
4. date of HIV-positive diagnosis;
5. seropositivity for syphilis and hepatitis B and C viruses;
6. number and classes of medications in use at last prescription in the medical file, and presence of potentially inappropriate medications for elderly patients based on the Portuguese versions of two active drug lists;^13^^,^^14^ and
7. estimated mean CD4 lymphocyte count based on the formulas: total lymphocyte count/6 and total lymphocyte count x 0.8 x 0.2 or 0.3.^15^

The present study was submitted to the institution’s Ethics Committee for Research on Humans on January 28, 2014, and was approved on March 6, 2014. The study has been registered as approved on the Brazil Platform (http://aplicacao.saude.gov.br/plataformabrasil) under CAAE 26867214.4.0000.5478.

## RESULTS

Four of the residents tested HIV-positive, i.e. approximately 1.0% of the total number of residents at the LTCI studied (total of 405 residents). All of these four individuals were men and their mean age was 71.2 ± 8.6 years (Table 1). Their mean length of stay at the LTCI was 74.2 ± 38.1 months and the mean length of time for which they had had an HIV-positive diagnosis (reached through standard HICC screening) was 24.5 ± 17.0 months. Two of them were found to be seropositive for syphilis, two for hepatitis B and one for hepatitis C. The estimated mean CD4 count was 341.1 ± 237.4/mm^3^.

**Table 1.**
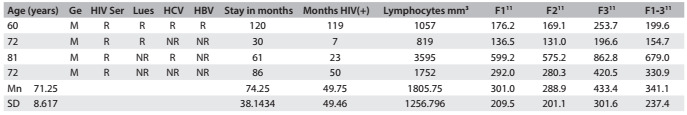
Human immunodeficiency virus-positive long-term care institution residents: general data, serological results, length of long-term care institution stay and lymphocyte count

Mn = mean; SD = standard deviation; Ge = gender; M = male; Ser = serology; R = reactive; NR = non-reactive; HIV = human immunodeficiency virus; Lues = syphilis; HCV = hepatitis C virus; HBV = hepatitis B virus; CD4 = T4 lymphocytes; F1^11^ = estimated mean CD4 lymphocyte count calculation using formula 1; F2^11^ = estimated mean CD4 lymphocyte count calculation using formula 2; F3^11^ = estimated mean CD4 lymphocyte count calculation using formula 3; F1-3^11^ = average.

Regarding underlying diseases, sequelae of stroke were present in three of these residents, while prostate cancer and dementia syndrome were found in one of them. The main drugs used were lamivudine, zidovudine, lopinavir, ritonavir, levothyroxine, omeprazole, ranitidine, lactulose and risperidone (Table 2). The mean number of medications taken was 10.5 ± 2.8 drugs per HIV-positive patient (range: 7-12 drugs per HIV-positive patient). These four patients were taking an average of 5.3 ± 1.5 antiretroviral drugs (all of them after they received the HIV diagnosis) and 5.3 ± 1.0 other drugs. The presence of potentially inappropriate medications for elderly individuals, based on the Portuguese versions of two active drug lists,^13^^,^^14^ was 0.7 ± 0.5 potentially inappropriate medications/patient (antihistamines and doxazosin).

**Table 2.**
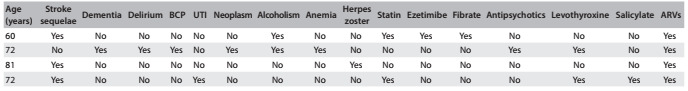
Human immunodeficiency virus-positive long-term care institution residents: age, underlying diseases and medications use

BCP = pneumonia; UTI = urinary tract infection; ARVs = antiretroviral drugs.

## DISCUSSION

Cases of AIDS among the elderly have been reported in Brazil over the last three decades. The proportion of elderly patients among all HIV-positive cases reported has been rising sharply due to the survival of carriers using antiretroviral therapy and the aging of the population.^2^^,^^6^^,^^7^^,^^8^^,^^9^ Consultation of the Brazilian literature revealed studies on various aspects of elderly patients with AIDS or who were HIV-positive, although HIV infection among LTCI residents in Brazil has not been fully investigated.^3^^,^^4^^,^^5^^,^^6^^,^^7^^,^^8^^,^^9^^,^^16^^,^^17^^,^^18^^,^^19^^,^^20^^,^^21^^,^^22^^,^^23^ Disparities in the definition of elderly among these published papers is also evident. Some of them used the age criterion of ≥ 50 years,^9^^,^^16^^,^^18^^,^^20^^,^^21^ while others adopted ≥ 60 years.^3^^,^^4^^,^^6^^,^^7^^,^^17^^,^^20^^,^^23^ The present study sample comprised residents ≥ 60 years of age.

One notable finding was that approximately 1.0% of the residents in the LTCI studied here were HIV-positive, which was higher than the previously estimated rates of 18.78 and 10.80 cases/100,000 males (1998 and 2010 respectively).^15^^,^^20^ The greater concentration of elderly patients with greater severity of physical and/or mental conditions in LTCIs, in comparison with the general elderly population, partially explains this discrepancy.^2^^,^^24^ The size of the present sample (four cases/405 beds) may also have contributed to this difference in rates. It should be noted, however, that these data were obtained through an active search using the HICC protocol of the LTCI studied, thus increasing the likelihood of detecting oligosymptomatic or asymptomatic cases. This explains the finding of an 81-year-old HIV-positive patient, an age group rarely reported.^6^^,^^17^^,^^23^

Previous studies have reported a predominance of males among elderly HIV-positive individuals.^6^^,^^7^^,^^9^^,^^18^^,^^20^ This phenomenon can be correlated with the lower survival rate observed among female elderly HIV-positive individuals.^17^ These findings may explain the result of only male cases in the present study, i.e. women do not survive long enough to be placed into LTCIs. Presuming that HIV infection preceded admission to the LTCI in the present study, the mean survival of these patients (74.2 ± 38.1 months or 6.2 ± 3.2 years) was longer than that reported in another study (3.4-4.6 years).^16^ This finding is also related to the fact that HIV infection was actively detected in these elderly individuals by applying the HICC screening protocol, rather than being diagnosed as a result of symptoms suggestive of AIDS. Given that 50.0% of HIV-positive elderly individuals survive no longer than six months after the first opportunist infection,^17^ it is clear that early detection was possible in these cases, thus changing the course of the syndrome and the survival of the infected residents at the LTCI analyzed.

The association of seropositivity for syphilis and/or hepatitis B and C viruses in the present study suggests that the HIV infection had been transmitted sexually. This finding has also been reported in other studies, and it has most notably occurred through heterosexual exposure.^6^^,^^16^^,^^17^^,^^18^^,^^20^^,^^23^

The estimated mean CD4 count (341.1 ± 237.4/mm^3^) mirrored what had previously been observed in 72.9% of HIV-infected individuals ≥ 60 years of age (≤ 350/mm^3^).^6^ Interestingly, the highest estimated mean CD4 count (679.0/mm^3^) in the present sample was found in an octogenarian patient. This elderly patient had had a short stay at the LTCI and presented few comorbidities (seropositivity for hepatitis C virus, stroke sequelae and Herpes zoster). This finding leads to speculation that the HIV infection in this age group indicates an active and/or slower-than-usual pattern of aging. It is also notable that the individual with the lowest estimated CD4 count (154.7/mm^3^) in this sample had had the shortest stay at the LTCI studied and presented a clinical picture of dementia syndrome with several other possible etiologies for his dementia overlapping with HIV (seropositivity for syphilis, alcoholism and hypothyroidism). Moreover, it can be seen that if the LTCI had not had active screening for HIV and syphilis-infected individuals, this would have implied a greater risk of contamination posed for the healthcare professionals at these facilities, with only partial treatment of the underlying causes of dementia, and the possibility that neurological infections might be overlooked.

The finding of stroke sequelae in three out of the four HIV-positive patients points towards two interpretations: chance (due to the small sample) or a clinical correlation warranting attention. Recent discussion has centered on whether HIV represents an independent risk factor for stroke.^25^^,^^26^^,^^27^^,^^28^ Some studies in the literature have investigated the association between HIV and stroke among children and young adults during the era preceding antiretroviral therapy and also between HIV and the risk of cerebrovascular events among individuals using these drugs.^25^^,^^26^^,^^27^^,^^28^ However, Hasse et al.^29^ reported a hazard ratio for stroke of 17.7, in a multivariable analysis on elderly HIV-positive individuals in Switzerland (confidence interval of 7.06 to 44.5), thus justifying attention to this clinical finding in the present study.

In the literature consulted, we did not find any studies reporting the association of HIV with prostate cancer that was seen in one patient of the present study. Nonetheless, the growing incidence of lethal solid tumors in individuals with HIV infection controlled by means of antiretroviral drugs has raised concerns.^30^^,^^31^

Pharmacokinetic and pharmacodynamic characteristics are known to be changed through the aging process,^32^^,^^33^ and polypharmacy is an additional contributory factor.^34^^,^^25^^,^^26^^,^^37^ Prescribing drugs for the elderly, with or without HIV, therefore remains a constant clinical challenge, given the high risks of drug-to-drug interactions, side effects and potentially inappropriate medications in this age group.^13^^,^^14^^,^^32^^,^^33^^,^^34^^,^^35^^,^^36^

Metabolic changes and cardiovascular diseases associated with the use of antiretroviral therapy are known, and these call for heightened vigilance among elderly patients. Despite the lack of specific consensus, use of statins and ezetimibe would have been justified in the cases of the present study because of the history of neurovascular events in three of the four patients, and also because of the presence of hypothyroidism in two patients and the need to take risperidone in another.^8^^,^^38^^,^^39^

The low presence of potentially inappropriate medications per patient was due to the regular checking for potentially inappropriate medications in prescriptions that was conducted by the LTCI. It is important to point out that neither of the two lists of potentially inappropriate medications indicates any antiretroviral drugs as potentially inappropriate for use among elderly patients.^13^^,^^14^

## CONCLUSION

HIV patients can be found in LTCIs: they are diagnosable through serological screening and treatable with antiretroviral drugs.

Specific investigation and treatment protocols for use among elderly individuals need to be developed in order to aid in the detection and therapeutic control of HIV.
